# Oropharyngeal and Sputum Microbiomes Are Similar Following Exacerbation of Chronic Obstructive Pulmonary Disease

**DOI:** 10.3389/fmicb.2017.01163

**Published:** 2017-06-22

**Authors:** Hai-Yue Liu, Shi-Yu Zhang, Wan-Ying Yang, Xiao-Fang Su, Yan He, Hong-Wei Zhou, Jin Su

**Affiliations:** ^1^Department of Environmental Health, School of Public Health, Southern Medical UniversityGuangzhou, China; ^2^State Key Laboratory of Organ Failure Research, Division of Laboratory Medicine, Zhujiang Hospital, Southern Medical UniversityGuangzhou, China; ^3^Chronic Airways Diseases Laboratory, Department of Respiratory and Critical Care Medicine, Nanfang Hospital, Southern Medical UniversityGuangzhou, China

**Keywords:** microbiome, 16S rRNA, ITS, AECOPD, sputum, oropharyngeal swab

## Abstract

Growing evidence suggests that the airway microbiota might be involved in acute exacerbation of chronic obstructive pulmonary disease (AECOPD). Understanding this relationship requires examination of a large-scale population for a long duration to accurately monitor changes in the microbiome. This type of longitudinal study requires an appropriate sampling strategy; two options are the collection of sputum or oropharyngeal swabs. Comparative analysis of the changes that occur in these two specimen types has not been previously performed. This observational study was conducted to explore oropharyngeal microbial community dynamics over time and to examine the relationship between oropharyngeal swabs and sputum. A total of 114 samples were collected from four patients suffering from severe AECOPD. Bacterial and fungal communities were evaluated using 16S rRNA and ITS sequencing. Inter-individual differences were found in bacterial community structure, but the core genera were shared by both sample types and included 32 lineages. Most of the core genera were members of the phyla Proteobacteria, Firmicutes and Ascomycota. Although the oropharyngeal samples showed higher bacterial alpha diversity, the two sample types generated rather similar taxonomic profiles. These results suggest that the sputum microbiome is remarkably similar to the oropharyngeal microbiome. Thus, oropharyngeal swabs can potentially be used instead of sputum samples for patients with exacerbation of COPD.

## Introduction

Chronic obstructive pulmonary disease (COPD) is a common and slowly progressive disease characterized by sustained and irreversible airflow limitations that lead to the gradual loss of lung function (Einarsson et al., 2016). As symptoms progress, patients experience severe shortness of breath and extreme dyspnea. This makes the collection of sputum samples challenging (Kim et al., 2011), as sputum examination requires the use of a high-permeability 5% saltwater spray, and patients often find it difficult to tolerate atomization, resulting in failure to induce sputum production for sampling. According to the World Health Organization, COPD will become the third most common cause of death globally by 2020 and will be the fifth most economically burdensome disease. The acute exacerbation of chronic obstructive pulmonary disease (AECOPD) is a major cause of this burden (Montes de Oca and Perez-Padilla, 2017).

Airway microbial communities associated with COPD have long been studied. AECOPD is mainly caused by infection with bacteria, viruses, and fungi, with more than 50% of cases being caused by bacterial infection (Sethi and Murphy, 2008). AECOPD is typically associated with the overgrowth of pathogenic bacteria, especially *Pseudomonas aeruginosa*, *Haemophilus influenzae*, *Streptococcus pneumoniae*, and *Moraxella catarrhalis*. In the airway, the presence of specific bacteria or fungi, such as *Moraxella* or *Aspergillus*, can increase the levels of inflammatory factors in the sputum and in turn exacerbate and increase the mortality associated with COPD (Hill et al., 2000; Huerta et al., 2014). Changes in airway microbial composition can also lead to exacerbation of COPD (Dy and Sethi, 2016). Therefore, studies of the changes that occur in the microbiome during AECOPD are crucial. A sputum-based longitudinal survey examining the function of the lung microbiome and its potential role in the disease etiology of AECOPD provides good examples to help understand the potential of the lung microbiome as a target for future respiratory therapeutics to manage COPD (Huang et al., 2014; Wang et al., 2016). Improved understanding of the microbial underpinnings of AECOPD will allow for the identification of therapeutic targets and the development of improved treatment options (Mammen and Sethi, 2016).

The anatomical structure of the airway is composed of a series of continuous channels: gas enters through the oral cavity or nose, passes through the pharynx and larynx, enters the trachea, and then gradually passes through the dendritic bronchi and bronchioles to the terminal bronchioles, respiratory bronchioles, and alveoli (Dmitrieva, 2013). Research has shown that the composition of the microbial community in the lower airways in healthy people is the same as that in the upper airways (Charlson et al., 2011). Additional research has further supported this opinion, showing similar microbial compositions in the upper and lower airways and also demonstrating that the lower airway possesses higher relative abundances of *Enterobacteriaceae* and *Haemophilus* (Morris et al., 2013). *Prevotella*, *Sphingomonas*, *Pseudomonas*, *Acinetobacter*, *Fusobacterium*, *Veillonella*, *Staphylococcus*, and *Streptococcus* are commonly detected bacteria in healthy individuals, as well as in COPD airway microbiota (Zakharkina et al., 2013). Further, a previous study has reported that the airway microbiome of COPD patients harbors more *Pseudomonas* spp. of Proteobacteria and *Lactobacillus* spp. of Firmicutes than that of healthy individuals (Park et al., 2014). The microbial community present in COPD patients has been clearly described based on research using either oropharyngeal swabs or sputum samples. This research has shown that the oropharyngeal microbiota of COPD patients is mainly composed of Proteobacteria, Bacteroidetes, Firmicutes, and Actinobacteria, similar to the sputum microbiota (Park et al., 2014; Huang and Boushey, 2015). In pulmonary fibrosis, tuberculosis, and other diseases, oropharyngeal samples can be used in place of sputum samples to determine the structure of the airway microbial community (Botero et al., 2014; Zemanick et al., 2015).

Growing evidence suggests that it is important to examine large-scale populations for longitudinal studies attempting to identify therapeutic targets (Huang et al., 2014; Wang et al., 2016). This type of longitudinal study requires an appropriate sampling strategy, and two optional strategies include the collection of sputum and oropharyngeal swabs. In many patients with extreme dyspnea, collecting sputum samples is challenging; in contrast, obtaining oropharyngeal swabs is a non-invasive and easy process. Moreover, a comparative analysis of the changes that occur in sputum and oropharyngeal samples has not been previously performed. This observational study was conducted to explore oropharyngeal microbial community dynamics over time and to examine the relationship between oropharyngeal and sputum samples.

## Materials and Methods

### Sample Collection

This study was carried out in accordance with the recommendations of the International Ethical Guidelines for Biomedical Research Involving Human Subjects and the ethics committee of Southern Medical University (Permit No. 2012-072), and all subjects provided written informed consent in accordance with the Declaration of Helsinki. The protocol was approved by the ethics committee of Southern Medical University. A total of 114 samples were collected at Nanfang Hospital, Southern Medical University (Guangzhou, China) between June 2012 and December 2012. The samples were collected from four patients with severe AECOPD during hospitalization stays of 14–17 days. All patients were male, and their age range was 68–83 years (**Table 1**). For sputum induction and processing, we used the guidelines suggested by the Task Force on Induced Sputum of the European Respiratory Society (Paggiaro et al., 2002; Vignola et al., 2002). The samples were immediately stored at -80°C for subsequent DNA extraction. Swabs were taken from the oropharyngeal wall.

**Table 1:** Clinical information for the four study subjects.

Subject	Age	Gender	FEV1%	Sputum culture results	Hospitalized days
A	83	Male	47.5	*Acinetobacter baumannii*	17
B	68	Male	23.3	None	15
C	80	Male	None	None	14
D	79	Male	31.2	NA	14

### Pulmonary Function (PF) Tests

Spirometry was performed using a Jaeger Masterscope spirometry system (Jaeger, Wuerzburg, Germany) according to the American Thoracic Society (ATS) guidelines (Miller et al., 2005). The forced expiratory volume in 1 s as a percentage of predicted (FEV1%) is considered an important diagnostic measurement of COPD. Commonly, FEV1% is used to measure the grade of COPD as follows: mild: FEV1% ≥ 80; moderate: 50 ≤ FEV1% < 80; severe: 30 ≤ FEV1% < 50; and very severe: FEV1% < 30. However, FEV1% alone may not adequately reflect a patient’s overall health status (van der Molen and Cazzola, 2012).

### Culture Method

Sputum culturing included homogenization with dithiothreitol and the plating of aliquots of serial dilutions on blood, chocolate, and MacConkey culture agars.

### DNA Extraction and PCR

Genomic DNA was extracted from each sample using a Total Nucleic Acid Extraction Kit (Bioeasy Technology, Inc., China) according to the manufacturer’s instructions. The 16S rRNA gene was amplified using barcoded V4 primers. The ITS gene was amplified using barcoded ITS1 primers and then purified and pooled as described in our earlier studies (He et al., 2013; Su et al., 2015). The 16S rRNA and ITS PCR products were sequenced at the Beijing Genomics Institute using paired-end sequencing on an Illumina HiSeq 2000 platform. For bacteria, PCR was performed with the bacterial-specific primers 514F-5′ GTGCCAGGMGCCGCGGTAA 3′ and 805R-5′ GGACTACHVGGGTWTCTAAT 3′. The reaction conditions were as follows: initialization at 94°C for 2 min, followed by 30 cycles of denaturation at 94°C for 30 s, annealing at 52°C for 30 s, and elongation at 72°C for 45 s, and a final elongation step at 72°C for 5 min. For fungi, PCR was performed with the fungal-specific primers ITSF 5′ CTTGGTCATTTAGAGGAAGTAA 3′ and ITSR 5′ GCTGCGTTCTTCATCGATGC 3′. The reaction conditions were as follows: initialization at 94°C for 15 min, followed by 5 cycles of denaturation at 95°C for 30 s, annealing at 50°C for 30 s, and elongation at 72°C for 60 s, and subsequently, 35 cycles of denaturation at 95°C for 30 s, annealing at 65°C for 30 s, and elongation at 72°C for 60 s, followed by a final elongation step at 72°C for 5 min.

### Sequence Processing and Analysis

The sequences were demultiplexed and quality filtered using the QIIME (Quantitative Insights Into Microbial Ecology) platform (1.9.1) (Caporaso et al., 2010). Sequencing was conducted to generate 100-bp paired-end reads using an Illumina HiSeq 2000 sequencer according to the manufacturer’s instructions. The Illumina sequencing quality report revealed that the sequence quality was relatively high for fragment sizes of up to 80 bp, with a sharp decrease in quality for larger fragments. Thus, we trimmed the raw sequences to 80 bp for each read pair. The sequences were then de-multiplexed, trimmed of barcodes and primers and filtered if they contained ambiguous bases or mismatches in the primer regions, according to the BIPES protocol (Zhou et al., 2011). The detailed protocol was as follows: first, we deleted the sequences with barcoded primers that contained ambiguous reads or mismatches in the primer region; then, we removed the primers and kept the remaining clean sequences of the 16S and ITS genes. Second, we removed any sequences with more than one mismatch within the 40–70 bp region at each end. Next, we used 30Ns to concatenate adjacent single-ended sequences for the subsequent sequence analyses, as our paired-end sequences did not extend to the V4 regions of the 16S rRNA gene (He et al., 2013). All the tools used in this study have been validated for use with gapped sequences. We screened for and removed chimeras using UCHIME in *de novo* mode (Edgar et al., 2011). The final high-quality sequence reads for the 16S and ITS genes were generated after the sequences were screened with UCHIME. The sequences were deposited in the European Nucleotide Archive (ENA), and the accession number was ERS1659093.

Subsequent analyses were implemented using the QIIME. The sequences were then clustered into operational taxonomic units (OTUs) using USEARCH with the default parameters and with the threshold distance set to 0.03 for 16S genes and 0 for the ITS genes. QIIME-derived reference-based alignments of representative sequences were performed using PyNAST with the Greengenes 13_8 database as the template file (Al-Hebshi et al., 2015). The Ribosome Database Project (RDP) algorithm was applied to classify representative 16S sequences into specific taxa using the default database (Wang et al., 2007). Representative ITS DNA gene sequences were classified using QIIME_ITS database as a reference (version information: sh_qiime_release_01.08.2015) (Caporaso et al., 2010). The biome data were filtered using the filter_otus_from_otu_table.py script with the parameter (-s 3) to remove low-abundance OTUs. Next, each 16S rRNA and ITS DNA gene sample was normalized to 1,000 sequences to avoid biases resulting from uneven sequencing depth among samples. All samples with less than 1,000 sequences and their paired (oropharyngeal swab/sputum) samples were not included in the later analysis.

To determine which sampling routine provided higher alpha diversity while controlling for patient and day effects, we used a simple up/down scoring system whereby on each subject-day, we recorded which sample had higher diversity. The overall results were then aggregated into a distribution and assessed using the Chi-square test. Adonis was used to estimate the dissimilarity of the microbial compositions between groups. Procrustes analysis was performed to determine whether the beta diversity results were similar between the oropharyngeal swabs and sputum samples. Weighted uniFrac distances based on the phylogenetic metric were used for these two analyses. Correlations of the core genera (representing >10% of the sequences in any sample) between the two sample types were assessed by Spearman correlation analysis. We identified differential features between the two groups using a linear discriminant analysis (LDA) effect size (LEfSe) method. The threshold cutoff values of the logarithmic LDA scores for identifying taxa that differed in abundance between the comparison groups were 3.5 for bacteria and 2.0 for fungi. To obtain OTU data for LEfSe analysis, we selected OTUs with a relative abundance of over 10% in any sample.

## Results

### Associations between Sample Sites, Individuals and Microbial Communities

**FIGURE 1:**
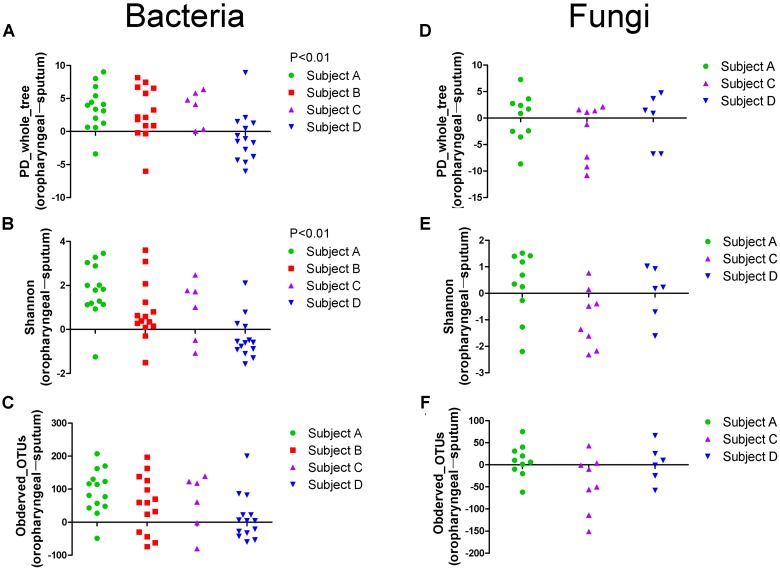
Scatter plot showing the alpha diversity measurements of the microbiota collected from the oropharyngeal and sputum samples. **(A)** PD_whole_tree, **(B)** Shannon and **(C)** Observed_OTUs indices of bacterial community. **(D)** PD_whole_tree, **(E)** Shannon and **(F)** Observed_OTUs indices of fungal community. For each patient, swab samples are compared to sputum samples from different days. Each point represents a difference between the oropharyngeal swab and sputum sample, as determined using a simple up/down scoring system whereby on each subject-day, the sample with the higher diversity was recorded (obtained using the Chi-square test). PD_whole_tree, Observed_OTUs, and Shannon indices were used as phylogenetic, richness, and evenness and richness metrics, respectively, to determine the diversities of the bacterial and fungal communities.

Alpha diversity measurements were used to assess variations in community structure among the microbiota collected in the oropharyngeal and sputum samples. Specifically, phylogenetic (PD_whole_tree), richness (Observed_OTUs), and evenness and richness metrics (Shannon) were employed to analyze community alpha diversity (**Figure 1**). We detected significant differences in the bacterial alpha diversity between the sputum and oropharyngeal samples for the PD_whole_tree and Shannon indices (*P* < 0.01). However, the comparisons did not reveal any significant differences in the fungal community compositions. These results indicated that the oropharyngeal samples had higher alpha diversity for bacterial communities.

**FIGURE 2:**
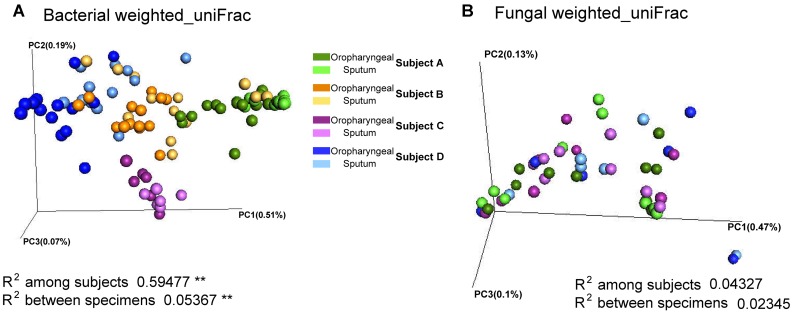
Beta diversity of sputum and oropharyngeal samples. **(A)** PCoA based on weighted_uniFrac distances for bacterial sequences obtained from Subjects A (green), B (orange), C (purple), and D (blue). **(B)** PCoA based on weighted_uniFrac distances for fungal sequences obtained from Subjects A (green), C (purple), and D (blue). The different sample types are indicated by the different shades (Adonis test, ^∗∗^*P* < 0.01).

Adonis analysis of the weighted_uniFrac distances performed using phylogenetic information revealed significant differences in the bacterial communities among individuals, indicating the presence of inter-individual differences in microbial composition (weighted_uniFrac distances, *P* < 0.01). In addition, the results revealed that the samples of the two specimen types had significantly different beta diversity results (weighted_uniFrac distances, *P* < 0.01), and greater inter-individual differences than inter-specimen differences were detected (**Figure 2**). In contrast, the results revealed non-significant differences in the fungal beta diversity results both between specimen types and among individuals. To visualize the microbial similarity and dissimilarity among the individuals and between the two sample types, PCoA was performed in the QIIME pipeline using the weighted_uniFrac distance. The results revealed broad overlap between the oropharyngeal and sputum samples, suggesting that both sample types shared similar bacterial/fungal communities. The samples from the four individuals were divided into four distinct clusters in terms of bacterial community composition.

### Microbial Composition Dynamics in Sputum and Oropharyngeal Samples

**FIGURE 3:**
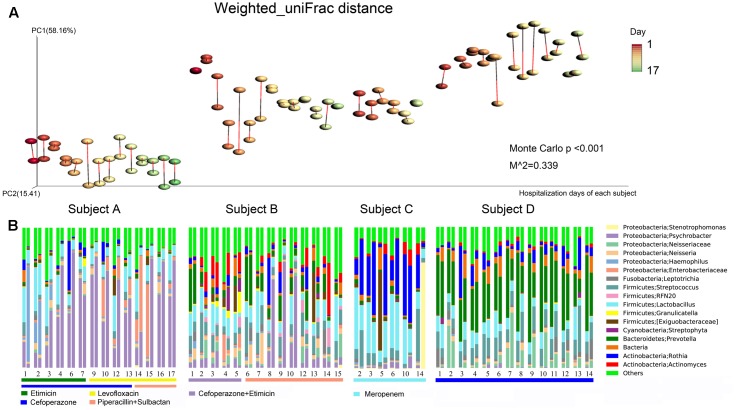
Bacterial community composition dynamics of the daily samples. **(A)** Procrustes analysis based on weighted_uniFrac distances was performed, which revealed the same beta diversity results for the oropharyngeal swabs and sputum samples for each subject-day. The points linked with bars represent data obtained on the same day but from two different species, red represents the oropharyngeal samples, and black represents the sputum samples in the edge-color pairs. **(B)** Relative abundances of the core bacterial genera in the daily samples. For the four subjects, the single-day paired bars are separated, with the oropharyngeal samples on the left and the sputum samples on the right. The rectangular bars show which classes of antibiotic each patient received during their hospital stay.

Large inter-individual differences in bacterial community structure were observed under different dynamic conditions. In Subject A, Proteobacteria (68.59%) and Firmicutes (20.52) were the predominant phyla, with *Psychrobacter* (43.76%) and *Lactobacillus* (13.77%) as the most prevalent genera. With regard to the dynamics in Subject A, the abundance of *Haemophilus* was high on the first 2 days and decreased starting on the 3rd day. In contrast, *Psychrobacter* sharply increased starting on the 3rd day, subsequently decreased on the 14th day and then increased again starting on the 16th day. The abundance of *Enterobacteriaceae* peaked on days 14 and 15. In addition, the abundance of *Psychrobacter* decreased quickly after piperacillin and sulbactam were added (**Figure 3B**).

In Subject B, Firmicutes (34.40%), Proteobacteria (27.71%), Bacteroidetes (14.50%), and Actinobacteria (13.47%) were the main phyla, and *Psychrobacter* (12.25%), *Lactobacillus* (11.21%), and *Prevotella* (10.00%) were the most common genera. The abundance of *Prevotella* was high on the 1st day, after which it decreased quickly and subsequently increased again starting on the 6th day. *Actinomyces* increased starting on the 2nd day, decreased on the 6th day, and then increased again on day 12. The abundance of *Psychrobacter* peaked on days 9 and 10, and it was only found to have a high abundance in the sputum samples. The abundances of *Psychrobacter*, *Actinomyces*, *Enterobacteriaceae*, and *Streptophyta* were decreased following the addition of piperacillin and sulbactam (**Figure 3B**).

In Subject C, Firmicutes (42.46%), Actinobacteria (35.88%) and Proteobacteria (13.90%) were the most common phyla, and *Rothia* (28.67%) and *Lactobacillus* (24.63%) were the predominant genera. In addition, the abundance of *Streptococcus* decreased whereas that of *Actinomyces* increased starting on the 10th day. In Subject D, Firmicutes (25.53%) and Proteobacteria (14.84%) constituted the majority of sequences, and *Prevotella* (34.77%) and *Streptococcus* (12.18%) were the most common genera (**Figure 3B**). In addition, we observed that the sputum and oropharyngeal sample dynamics were similar.

Procrustes analysis based on the bacterial weighted_uniFrac distances of the time series data revealed that the beta diversity results were the same for both sample types (**Figure 3A**). These findings further demonstrated that the oropharyngeal swab and sputum sample dynamics were similar, indicating that they had similar bacterial community structures.

**FIGURE 4:**
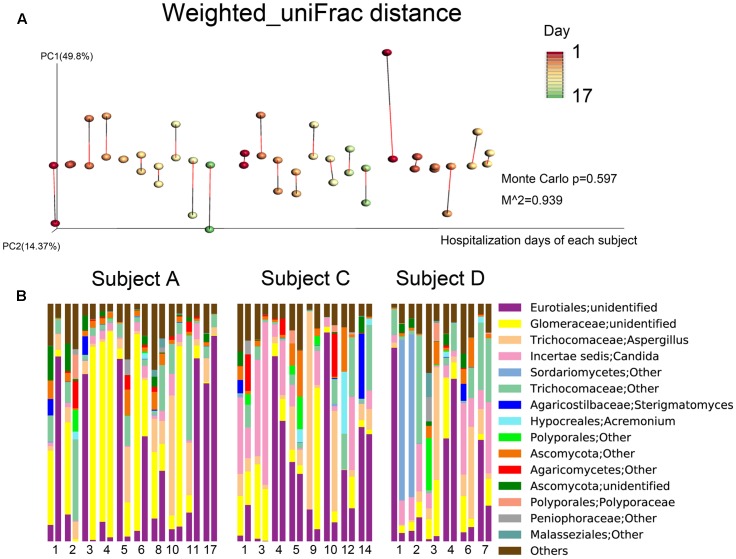
Fungal community composition dynamics of the daily samples. **(A)** Procrustes analysis based on weighted_uniFrac distances was performed, which revealed the same beta diversity results for the oropharyngeal swabs and sputum samples for each subject-day. The points linked with bars represent data obtained on the same day but from two different species, red represents the oropharyngeal samples, and green represents the sputum samples in the edge-color pairs. **(B)** Relative abundances of the core fungal genera in the daily samples. For the three subjects, the single-day paired bars are separated, with the oropharyngeal samples on the left and the sputum samples on the right.

In terms of fungal composition, the inter-individual and inter-specimen differences were small (**Figure 2B**). Meanwhile, Procrustes analysis based on the fungal weighted_uniFrac distances of the time series data did not revealed a same beta diversity for both sample types (**Figure 4A**). Ascomycota was the most predominant phylum among all the samples (72.59%), followed by Glomeromycota (19.89%) and Basidiomycota (9.66%). These three phyla represented over 99% of all the fungi sequences. At the genus level, *Eurotiales|unidentified* (27.91%), *Glomeraceae|unidentified* (18.33%), *Aspergillus* (10.25%), *Candida* (9.14%), and *Trichocomaceae|Other* (9.13%) were the most frequently identified fungi for the three individuals. In addition, the microbial compositions of the sputum and oropharyngeal samples collected on the same day and from the same subject were found to be very similar on most of the observation days (**Figure 4B**).

We further demonstrated that most sequences were shared between the two specimen types and that the unique taxonomic units and OTUs found in the sputum and oropharyngeal samples had relative abundances of less than 0.001. Among the 32 core genera (defined as the taxa representing more than 10% of the relative abundance in any sample), 16 were successfully assigned to a specific genus, including: *Psychrobacter, Stenotrophomonas, Haemophilus*, and *Neisseria* from Proteobacteria; *Streptococcus*, *Granulicatella*, and *Lactobacillus* from Firmicutes; *Actinomyces* and *Rothia* from Actinobacteria; *Leptotrichia* from Fusobacteria; *Prevotella* from Bacteroidetes; *Streptophyta* from Cyanobacteria; *Aspergillus*, *Acremonium*, and *Candida* from Ascomycota; and *Sterigmatomyces* from Basidiomycota. We further examined the correlations between the core genera in the paired samples. The results showed that 12 out of 17 of the core bacterial genera were significantly correlated between the two specimens (**Table 2**). Furthermore, we found no significant correlations between the sample types in terms of the core fungal genera.

**Table 2:** Correlations between the core genera in the paired samples (>10% sequences in any sample).

Genus	Spearman’s rank correlation coefficient (rs)	Significance (*p*)
Proteobacteria|*Psychrobacter*	0.782	0.000
Proteobacteria|*Stenotrophomonas*	0.360	0.012
Proteobacteria|*Haemophilus*	0.536	0.000
Bacteroidetes|*Prevotella*	0.822	0.000
Proteobacteria|*Enterobacteriaceae;g_*	0.698	0.000
Firmicutes|*Exiguobacteraceae;g_*	0.321	0.026
Actinobacteria|*Rothia*	0.446	0.001
Proteobacteria|*Neisseriaceae;g_*	0.642	0.000
Actinobacteria|*Actinomyces*	0.663	0.000
Firmicutes|*Streptococcus*	0.662	0.000
Firmicutes|*Granulicatella*	0.363	0.011
Firmicutes|*RFN20*	0.816	0.000

### Differences in Microbiota Composition between the Oropharyngeal and Sputum Samples

**FIGURE 5:**
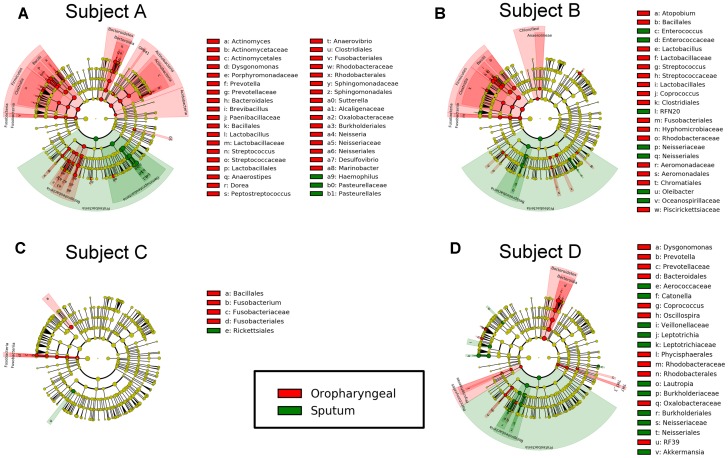
Differential features in bacteria selected via LEfSe between the sputum and oropharyngeal samples from each individual. **(A)** For Subject A, **(B)** for Subject B, **(C)** for Subject C, **(D)** for Subject D. Red represents the oropharyngeal samples, and green represents the sputum samples.

We performed LEfSe analysis for each individual and identified several similar discriminating features among the different individuals. For bacteria, the oropharyngeal samples were enriched with Bacteroidetes, i.e., *Prevotella* and *Dysgonomonas*; Firmicutes, i.e., *Lactobacillus*, *Coprococcus*, and *Streptococcus*; and Fusobacteria, i.e., *Fusobacteriales*. In contrast, the sputum samples contained a high prevalence of Proteobacteria. In addition, several distinct features were only detected in a single individual. Genera showing increased abundances in the oropharyngeal samples included *Actinomyces*, *Brevibacillus*, *Peptostreptococcus*, *Anaerovibrio*, *Sutterella*, *Neisseria*, *Desulfovibrio*, *Atopobium*, *Fusobacterium*, and *Oscillospira*. In contrast, genera with elevated abundances in the sputum samples included *Haemophilus*, *Enterococcus*, *RFN20*, *Oleibacter*, *Catonella*, *Leptotrichia*, *Lautropia*, and *Akkermansia*. Shared distinct features were more frequently observed in the oropharyngeal swabs from the different individuals, whereas differences in the distinct features were more commonly detected in the sputum samples from the individuals (**Figure 5**). LEfSe analysis, performed using the OTUs for further examination, revealed that *Prevotella melaninogenica* and *Lactobacillus iners* were the most abundant species in the oropharyngeal swabs (**Figure 7**).

**FIGURE 6:**
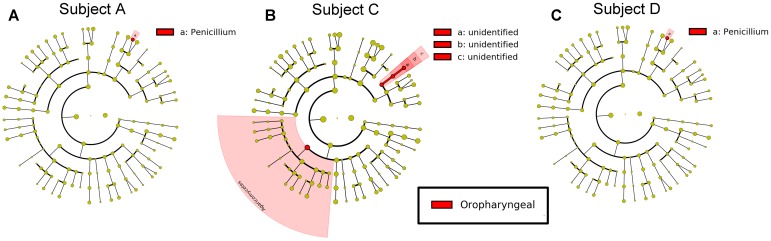
Differential features in fungi selected via LEfSe between the sputum and oropharyngeal samples from each individual. **(A)** For Subject A, **(B)** for Subject C, **(C)** for Subject D. Red represents the oropharyngeal samples.

**FIGURE 7:**
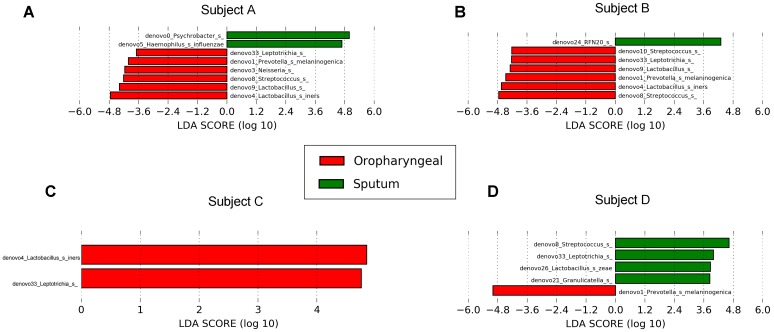
Differential core OTUs with more than 10% relative abundance in bacteria selected via LEfSe between the sputum and oropharyngeal samples from each individual. **(A)** For Subject A, **(B)** for Subject B, **(C)** for Subject C, **(D)** for Subject D. Red represents the oropharyngeal samples, and green represents the sputum samples.

For fungi, a LEfSe comparison of the oropharyngeal and sputum samples showed that *Penicillium* existed at a high abundance in the oropharyngeal samples (**Figure 6**). The results of our study demonstrated that the microbial community structures of these two ecological niches were much more similar for fungi than for bacteria.

## Discussion

Acute exacerbation of chronic obstructive pulmonary disease is a chronic progressive disease characterized by shortness of breath, expectoration, and the gradual development of severe dyspnea. It is difficult for some patients with severe to very severe AECOPD to expel sputum, necessitating respiratory support (Kim et al., 2011). Sputum-based longitudinal airway microbiome studies have been performed for the deep exploration of therapeutic targets and the development of improved treatment options (Wang et al., 2016). The upper airway is considered the beginning of the microbiological community of the body, including bacteria and fungi (Delhaes et al., 2012). Recent studies have indicated that oropharynx communities vary in terms of relative abundances and that they resemble those in sputum samples, consistent with the results of this study (Botero et al., 2014; Zemanick et al., 2015). In this work, we first examined the daily microbiota dynamics present in oropharyngeal swabs and sputum samples collected from COPD patients. We discovered that both specimen types exhibited similar microbial compositions and that the dynamics of these compositions were largely consistent.

The initial comparison showed that the oropharyngeal samples had higher diversity than the sputum samples in AECOPD, in contrast with previous studies reporting no differences in diversity between these two specimen types (Zemanick et al., 2015). This discrepancy may reflect influences of antibiotic use. The observed beta diversity in the bacterial/fungal communities according to the day of hospitalization indicates that the differences between the individuals were far greater than the differences between the sputum and oropharyngeal samples. Furthermore, the bacterial communities were found to have characteristic structures in different individuals (Wang et al., 2016). The fungal community structure also showed that the differences between individuals were greater than the differences between sampling points. Previous research on microbial communities in oropharyngeal swab and sputum samples has demonstrated high abundances of *Haemophilus*, *Prevotella*, and *Streptococcus* (Huang and Boushey, 2015; Ogorodova et al., 2015). These common genera were also detected in our study, with high linear correlations in the paired samples. Further, in this study, the bacterial community compositions were found to vary identically in each paired sample analyzed. Moreover, each microbiome with an abundance of more than 0.001 was shared between the sputum and oropharyngeal samples.

It has previously been shown that Proteobacteria, Firmicutes, and Actinomycetes account for a large proportion of the species present in the lower respiratory tract of moderate to severe COPD patients (44, 16, and 13%, respectively), which is similar to our results regarding these three phyla (Garcia-Nunez et al., 2014). According to previous studies, the relative abundance of *Actinomycetes* was 2% in the lower respiratory tract of COPD patients and 10% in the lower respiratory tract of healthy individuals. Similarly, the relative abundances of *Prevotella* in COPD patients and healthy individuals were 4.2 and 13.42%, respectively (Park et al., 2014; Einarsson et al., 2016). Our results for *Actinomycetes* in the oropharyngeal and sputum samples were similar to those of previous studies. In addition, the microbial composition of the sputum and oropharyngeal samples was consistent between pairs of samples collected each day. In our study, *Psychrobacter*, *Lactobacillus*, *Rothia*, *Prevotella*, *Neisseria*, *Streptococcus*, *Haemophilus*, *Actinomyces*, *Leptotrichia*, and *Aspergillus* were observed to have high relative abundances in both the sputum and oropharyngeal samples in individuals with severe AECOPD. According to previous reports, oropharyngeal and sputum samples from COPD patients have high abundances of *Haemophilus*, *Streptococcus*, and *Prevotella*, whereas sputum samples from COPD patients have high abundances of *Psychrobacter* and *Neisseria* (Aguirre et al., 2015; Huang and Boushey, 2015; Ogorodova et al., 2015; Wang et al., 2016). In previous reports, *Aspergillus* has been found to be associated with severe AECOPD, and its presence can lead to decreased lung function in affected patients (Morris et al., 2008; Barberan and Mensa, 2014). We compared the correlation between oropharyngeal and sputum samples for taxa with relative abundances above 10% and found that the two sample types were highly correlated with regard to the structure of these major taxa. Indeed, 70.59% of the bacteria had a high statistical correlation. This further supports the finding that the two sample types are consistent in describing the microbial community of AECOPD patients.

For bacteria, *Prevotella*, *Dysgonomonas*, *Lactobacillus*, *Coprococcus*, *Streptococcus*, and *Fusobacteriales* from Bacteroidetes, Firmicutes, and Fusobacteria accounted for most of the increased taxonomic abundances in the oropharyngeal samples, which is consistent with the findings of previous studies. Among these, *Streptococcus*, *Lactobacillus*, and *Prevotella* are the most common genera in the oral cavity (Zaura et al., 2009). *Haemophilus* and *Lautropia* were significantly more abundant in the sputum samples than in the oropharyngeal samples in our study. In addition, healthy lower airways have been reported to possess a higher relative abundance of *Haemophilus* than upper airways (Morris et al., 2013). At the OTU level, *P. melaninogenica* and *L. iners* were significantly increased in the oropharyngeal samples. *P. melaninogenica* typically colonizes oral cavities and is transferred from maternal saliva to children shortly after birth (Kononen et al., 1994). Similarly, *L. iners* is a common oral cavity bacteria (Anderson et al., 2014). The presence of resident bacteria in the oral cavity may reduce the proportion of major pathogens in AECOPD to a certain extent.

Subject A was treated with etimicin and cefoperazone, an aminoglycoside and β-lactam antibiotic, respectively. These antibiotics are sensitive to most Gram-positive and negative bacteria, especially *P. aeruginosa*, *H. influenza*, and *S. pneumoniae*. However, these antibiotics may fail to kill β-lactamase-producing bacteria; thus, they were replaced with piperacillin and sulbactam after 14 days for this subject. After the antibiotics were switched, we found a decreased abundance of *Psychrobacter*. This result is in agreement with previous reports showing that β-lactamase is secreted by *Psychrobacter* (Feller et al., 1995, 1997). The antibiotic treatment regimens were similar for Subjects B and A. We observed that the relative abundance of *Prevotella* was the lowest after the 1st day of treatment. In addition, the combined treatment with piperacillin and sulbactam inhibited *Psychrobacter*, *Actinomyces*, *Enterobacteriaceae*, and *Streptophyta*. These results showed that the distinctive spectrum of different antibiotics and antibiotic combination were relatively more efficient at killing the anti-pathogenic bacteria (Chandrasekaran et al., 2016). For the other subjects, meropenem or cefoperazone was administered based on their clinical conditions. Subject C displayed an increased abundance of *Stenotrophomonas*, which might have been caused by drug fast to the antibiotics (Zhao et al., 2017).

Previous studies have reported high levels of *Moraxella*, *Staphylococcus*, and *Pseudomonas* in COPD patients (Zakharkina et al., 2013), but this was not observed in our study. Most likely, this discrepancy occurred mainly because the included patients were being treated with antibiotics. It has also been shown that the V4 protocol used does not efficiently detect *Staphylococcus*, which might explain the low abundance of *Staphylococcus* observed in this study (Kong, 2016). The main microbial communities of the four included subjects were found to be quite unique. We examined four individuals to perform comprehensive day-to-day comparisons between sputum and oropharyngeal microbial communities for increased accuracy. In this study, we analyzed the ITS data with the classification of each individual sequence to generate a classification-based OTU table (with clustering at a threshold distance of 0), in contrast with a previous study with clustering at a threshold distance of 3 (Sokol et al., 2015). In this study, clustering at 0% difference increased the fungal signals and showed a more meaningful pattern in the fungal signals. Thus, we think clustering at 0% difference is suitable for analysis of our ITS data.

## Conclusion

The airway microbial communities were similar in terms of the main phylum and genus compositions, and the oropharyngeal swab and sputum sample dynamics were similar, demonstrating similar bacterial community structures. For oral bacterial colonization, the oropharyngeal bacterial community diversity was higher than that observed in the sputum samples. These results suggest that the sputum microbiome is remarkably similar to the oropharyngeal microbiome; thus, oropharyngeal swabs can potentially be used instead of sputum samples for patients with exacerbation of COPD.

## Author Contributions

Substantial contributions to the conception or design of the work or the acquisition, analysis, or interpretation of data for the work: H-YL, S-YZ, W-YY, H-WZ, YH, X-FS and JS. Drafting the work or revising it critically for important intellectual content: H-WZ, YH, X-FS, JS, H-YL, S-YZ and W-YY. Final approval of the version to be published: H-WZ, JS, H-YL, YH, X-FS, S-YZ and W-YY. Agreement to be accountable for all aspects of the work in ensuring that questions related to the accuracy or integrity of any part of the work are appropriately investigated and resolved: W-YY, H-YL, S-YZ, H-WZ, YH, X-FS, and JS.

## Conflict of Interest Statement

The authors declare that the research was conducted in the absence of any commercial or financial relationships that could be construed as a potential conflict of interest.

## References

[B1] Aguirre E. Galiana A. Mira A. Guardiola R. Sanchez-Guillen L. Garcia-Pachon E. (2015). Analysis of microbiota in stable patients with chronic obstructive pulmonary disease. *APMIS* 123 427–432. 10.1111/apm.12363 25858184

[B2] Al-Hebshi N. N. Nasher A. T. Idris A. M. Chen T. (2015). Robust species taxonomy assignment algorithm for 16S rRNA NGS reads: application to oral carcinoma samples. *J. Oral Microbiol.* 7 28934–28942. 10.3402/jom.v7.28934 26426306 PMC4590409

[B3] Anderson A. C. Sanunu M. Schneider C. Clad A. Karygianni L. Hellwig E. (2014). Rapid species-level identification of vaginal and oral lactobacilli using MALDI-TOF MS analysis and 16S rDNA sequencing. *BMC Microbiol.* 14:312 10.1186/s12866-014-0312-5 PMC4272787 25495549

[B4] Barberan J. Mensa J. (2014). Invasive pulmonary aspergillosis in patients with chronic obstructive pulmonary disease. *Rev. Iberoam. Micol.* 31 237–241. 10.1016/j.riam.2014.07.004 25481431

[B5] Botero L. E. Delgado-Serrano L. Cepeda M. L. Bustos J. R. Anzola J. M. Del Portillo P. (2014). Respiratory tract clinical sample selection for microbiota analysis in patients with pulmonary tuberculosis. *Microbiome* 2 29–35. 10.1186/2049-2618-2-29 25225609 PMC4164332

[B6] Caporaso J. G. Kuczynski J. Stombaugh J. Bittinger K. Bushman F. D. Costello E. K. (2010). QIIME allows analysis of high-throughput community sequencing data. *Nat. Methods* 7 335–336. 10.1038/nmeth.f.303 20383131 PMC3156573

[B7] Chandrasekaran S. Cokol-Cakmak M. Sahin N. Yilancioglu K. Kazan H. Collins J. J. (2016). Chemogenomics and orthology-based design of antibiotic combination therapies. *Mol. Syst. Biol.* 12 872–893. 10.15252/msb.20156777 27222539 PMC5289223

[B8] Charlson E. S. Bittinger K. Haas A. R. Fitzgerald A. S. Frank I. Yadav A. (2011). Topographical continuity of bacterial populations in the healthy human respiratory tract. *Am. J. Respir. Crit. Care Med.* 184 957–963. 10.1164/rccm.201104-0655OC 21680950 PMC3208663

[B9] Delhaes L. Monchy S. Frealle E. Hubans C. Salleron J. Leroy S. (2012). The airway microbiota in cystic fibrosis: a complex fungal and bacterial community–implications for therapeutic management. *PLoS ONE* 7:e36313 10.1371/journal.pone.0036313 PMC3338676 22558432

[B10] Dmitrieva L. I. (2013). Diagnostic algorithm and quality indicator for the radiodiagnosis of chronic obstructive pulmonary disease on the principles of evidence-based medicine. *Vestn. Rentgenol. Radiol.* 196 50–56. 23700928

[B11] Dy R. Sethi S. (2016). The lung microbiome and exacerbations of COPD. *Curr. Opin. Pulm. Med.* 22 196–202. 10.1097/MCP.0000000000000268 26964078

[B12] Edgar R. C. Haas B. J. Clemente J. C. Quince C. Knight R. (2011). UCHIME improves sensitivity and speed of chimera detection. *Bioinformatics* 27 2194–2200. 10.1093/bioinformatics/btr381 21700674 PMC3150044

[B13] Einarsson G. G. Comer D. M. Mcilreavey L. Parkhill J. Ennis M. Tunney M. M. (2016). Community dynamics and the lower airway microbiota in stable chronic obstructive pulmonary disease, smokers and healthy non-smokers. *Thorax* 71 795–803. 10.1136/thoraxjnl-2015-207235 27146202

[B14] Feller G. Sonnet P. Gerday C. (1995). The beta-lactamase secreted by the antarctic psychrophile *Psychrobacter immobilis* A8. *Appl. Environ. Microbiol.* 61 4474–4476. 8534113 10.1128/aem.61.12.4474-4476.1995 PMC167757

[B15] Feller G. Zekhnini Z. Lamotte-Brasseur J. Gerday C. (1997). Enzymes from cold-adapted microorganisms. The class C beta-lactamase from the antarctic psychrophile *Psychrobacter immobilis* A5. *Eur. J. Biochem.* 244 186–191. 10.1111/j.1432-1033.1997.00186.x 9063463

[B16] Garcia-Nunez M. Millares L. Pomares X. Ferrari R. Perez-Brocal V. Gallego M. (2014). Severity-related changes of bronchial microbiome in chronic obstructive pulmonary disease. *J. Clin. Microbiol.* 52 4217–4223. 10.1128/JCM.01967-14 25253795 PMC4313290

[B17] He Y. Zhou B. J. Deng G. H. Jiang X. T. Zhang H. Zhou H. W. (2013). Comparison of microbial diversity determined with the same variable tag sequence extracted from two different PCR amplicons. *BMC Microbiol.* 13:208 10.1186/1471-2180-13-208 PMC3848352 24034943

[B18] Hill A. T. Campbell E. J. Hill S. L. Bayley D. L. Stockley R. A. (2000). Association between airway bacterial load and markers of airway inflammation in patients with stable chronic bronchitis. *Am. J. Med.* 109 288–295. 10.1016/S0002-9343(00)00507-6 10996579

[B19] Huang Y. J. Boushey H. A. (2015). The sputum microbiome in chronic obstructive pulmonary disease exacerbations. *Ann. Am. Thorac. Soc.* 12(Suppl. 2), S176–S180. 10.1513/AnnalsATS.201506-319AW 26595736 PMC4722839

[B20] Huang Y. J. Sethi S. Murphy T. Nariya S. Boushey H. A. Lynch S. V. (2014). Airway microbiome dynamics in exacerbations of chronic obstructive pulmonary disease. *J. Clin. Microbiol.* 52 2813–2823. 10.1128/JCM.00035-14 24850358 PMC4136157

[B21] Huerta A. Soler N. Esperatti M. Guerrero M. Menendez R. Gimeno A. (2014). Importance of *Aspergillus* spp. isolation in Acute exacerbations of severe COPD: prevalence, factors and follow-up: the FUNGI-COPD study. *Respir Res.* 15 17–25. 10.1186/1465-9921-15-17 24517318 PMC3996133

[B22] Kim V. Garfield J. L. Grabianowski C. L. Krahnke J. S. Gaughan J. P. Jacobs M. R. (2011). The effect of chronic sputum production on respiratory symptoms in severe COPD. *COPD* 8 114–120. 10.3109/15412555.2011.558546 21495839

[B23] Kong H. H. (2016). Details matter: designing skin microbiome studies. *J. Invest. Dermatol.* 136 900–902. 10.1016/j.jid.2016.03.004 27107375 PMC5482528

[B24] Kononen E. Saarela M. Karjalainen J. Jousimies-Somer H. Alaluusua S. Asikainen S. (1994). Transmission of oral *Prevotella melaninogenica* between a mother and her young child. *Oral Microbiol. Immunol.* 9 310–314. 10.1111/j.1399-302X.1994.tb00077.x 7808775

[B25] Mammen M. J. Sethi S. (2016). COPD and the microbiome. *Respirology* 21 590–599. 10.1111/resp.12732 26852737

[B26] Miller M. R. Hankinson J. Brusasco V. Burgos F. Casaburi R. Coates A. (2005). Standardisation of spirometry. *Eur. Respir. J.* 26 319–338. 10.1183/09031936.05.00034805 16055882

[B27] Montes de Oca M. Perez-Padilla R. (2017). Global initiative for chronic obstructive lung disease (GOLD)-2017: the ALAT perspective. *Arch. Bronconeumol.* 53 87–88. 10.1016/j.arbr.2017.01.016 28222935

[B28] Morris A. Beck J. M. Schloss P. D. Campbell T. B. Crothers K. Curtis J. L. (2013). Comparison of the respiratory microbiome in healthy nonsmokers and smokers. *Am. J. Respir. Crit. Care Med.* 187 1067–1075. 10.1164/rccm.201210-1913OC 23491408 PMC3734620

[B29] Morris A. Sciurba F. C. Norris K. A. (2008). Pneumocystis: a novel pathogen in chronic obstructive pulmonary disease? *COPD* 5 43–51. 10.1080/15412550701817656 18259974 PMC2602875

[B30] Ogorodova L. M. Fedosenko S. V. Popenko A. S. Petrov V. A. Tyakht A. V. Saltykova I. V. (2015). Comparison study of oropharyngeal microbiota in case of bronchial asthma and chronic obstructive pulmonary disease in different severity levels. *Vestn. Ross. Akad. Med. Nauk* 70 669–678. 10.15690/vramn532 27093794

[B31] Paggiaro P. L. Chanez P. Holz O. Ind P. W. Djukanovic R. Maestrelli P. (2002). Sputum induction. *Eur. Respir. J. Suppl.* 37 3s–8s. 12361361 10.1183/09031936.02.00000302

[B32] Park H. Shin J. W. Park S. G. Kim W. (2014). Microbial communities in the upper respiratory tract of patients with asthma and chronic obstructive pulmonary disease. *PLoS ONE* 9:e109710 10.1371/journal.pone.0109710 PMC4199592 25329665

[B33] Sethi S. Murphy T. F. (2008). Infection in the pathogenesis and course of chronic obstructive pulmonary disease. *N. Engl. J. Med.* 359 2355–2365. 10.1056/NEJMra0800353 19038881

[B34] Sokol H. Leducq V. Aschard H. Pham H. P. Jegou S. Landman C. (2015). Fungal microbiota dysbiosis in IBD. *Gut* 66 1039–1048. 10.1136/gutjnl-2015-310746 PMC5532459 26843508

[B35] Su J. Liu H. Y. Tan X. L. Ji Y. Jiang Y. X. Prabhakar M. (2015). Sputum bacterial and fungal dynamics during exacerbations of severe COPD. *PLoS ONE* 10:e0130736 10.1371/journal.pone.0130736 PMC4493005 26147303

[B36] van der Molen T. Cazzola M. (2012). Beyond lung function in COPD management: effectiveness of LABA/LAMA combination therapy on patient-centred outcomes. *Prim. Care Respir. J.* 21 101–108. 10.4104/pcrj.2011.00102 22222945 PMC6547888

[B37] Vignola A. M. Rennar S. I. Hargreave F. E. Fah J. V. Bonsignore M. R. Djukanovic R. (2002). Standardised methodology of sputum induction and processing. Future directions. *Eur. Respir. J. Suppl.* 37 51s–55s. 12361365

[B38] Wang Q. Garrity G. M. Tiedje J. M. Cole J. R. (2007). Naive Bayesian classifier for rapid assignment of rRNA sequences into the new bacterial taxonomy. *Appl. Environ. Microbiol.* 73 5261–5267. 10.1128/AEM.00062-07 17586664 PMC1950982

[B39] Wang Z. Bafadhel M. Haldar K. Spivak A. Mayhew D. Miller B. E. (2016). Lung microbiome dynamics in COPD exacerbations. *Eur. Respir. J.* 47 1082–1092. 10.1183/13993003.01406-2015 26917613

[B40] Zakharkina T. Heinzel E. Koczulla R. A. Greulich T. Rentz K. Pauling J. K. (2013). Analysis of the airway microbiota of healthy individuals and patients with chronic obstructive pulmonary disease by T-RFLP and clone sequencing. *PLoS ONE* 8:e68302 10.1371/journal.pone.0068302 PMC3706416 23874580

[B41] Zaura E. Keijser B. J. Huse S. M. Crielaard W. (2009). Defining the healthy “core microbiome” of oral microbial communities. *BMC Microbiol.* 9:259 10.1186/1471-2180-9-259 PMC2805672 20003481

[B42] Zemanick E. T. Wagner B. D. Robertson C. E. Stevens M. J. Szefler S. J. Accurso F. J. (2015). Assessment of airway microbiota and inflammation in cystic fibrosis using multiple sampling methods. *Ann. Am. Thorac. Soc.* 12 221–229. 10.1513/AnnalsATS.201407-310OC 25474078 PMC4342834

[B43] Zhao S. Yang L. Liu H. Gao F. (2017). *Stenotrophomonas maltophilia* in a university hospital of traditional Chinese medicine: molecular epidemiology and antimicrobial resistance. *J. Hosp. Infect.* 10.1016/j.jhin.2017.04.001 28502480

[B44] Zhou H. W. Li D. F. Tam N. F. Jiang X. T. Zhang H. Sheng H. F. (2011). BIPES, a cost-effective high-throughput method for assessing microbial diversity. *ISME J.* 5 741–749. 10.1038/ismej.2010.160 20962877 PMC3105743

